# Rapid in-country sequencing of whole virus genomes to inform rabies elimination programmes

**DOI:** 10.12688/wellcomeopenres.15518.2

**Published:** 2020-05-19

**Authors:** Kirstyn Brunker, Gurdeep Jaswant, S.M. Thumbi, Kennedy Lushasi, Ahmed Lugelo, Anna M. Czupryna, Fred Ade, Gati Wambura, Veronicah Chuchu, Rachel Steenson, Chanasa Ngeleja, Criselda Bautista, Daria L. Manalo, Ma. Ricci R. Gomez, Maria Yna Joyce V. Chu, Mary Elizabeth Miranda, Maya Kamat, Kristyna Rysava, Jason Espineda, Eva Angelica V. Silo, Ariane Mae Aringo, Rona P. Bernales, Florencio F. Adonay, Michael J. Tildesley, Denise A. Marston, Daisy L. Jennings, Anthony R. Fooks, Wenlong Zhu, Luke W. Meredith, Sarah C. Hill, Radoslaw Poplawski, Robert J. Gifford, Joshua B. Singer, Mathew Maturi, Athman Mwatondo, Roman Biek, Katie Hampson

**Affiliations:** 1 Institute of Biodiversity, Animal Health and Comparative Medicine, University of Glasgow, Glasgow, G12 8QQ, UK; 2 The Boyd Orr Centre for Population and Ecosystem Health, University of Glasgow, Glasgow, G12 8QQ, UK; 3 University of Nairobi Institute of Tropical and Infectious Diseases (UNITID), Nairobi, Kenya; 4 Center for Global Health Research, Kenya Medical Research Institute, Nairobi, Kenya; 5 Paul G. Allen School for Global Animal Health, Washington State University, Pullman, WA, USA; 6 Ifakara Health Institute, Ifakara, Tanzania; 7 Department of Veterinary Medicine and Public Health, Sokoine University of Agriculture, Morogoro, Tanzania; 8 Tanzania Veterinary Laboratory Agency, Ministry of Livestock and Fisheries Development, Dar es Salaam, Tanzania; 9 Research Institute for Tropical Medicine (RITM), Manilla, Philippines; 10 Field Epidemiology Training Program Alumni Foundation (FETPAFI), Manilla, Philippines; 11 The Zeeman Institute for Systems Biology & Infectious Disease Epidemiology Research, School of Life Sciences and Mathematical Institute, University of Warwick, Coventry, UK; 12 Department of Agriculture Regional Field Office 5, Regional Animal Disease, Diagnostic Laboratory, Cabangan, Camalig, Albay, Philippines; 13 Albay Veterinary Office, Provincial Government of Albay, Albay Farmers' Bounty Village, Cabangan, Camalig, Albay, Philippines; 14 Wildlife Zoonoses & Vector-Borne Diseases Research Group, Animal and Plant Health Agency (APHA), Weybridge, UK; 15 Institute of Infection and Global Health,, University of Liverpool, Liverpool, UK; 16 Department of Pathology, University of Cambridge, Cambridge, UK; 17 University of Oxford, Oxford, UK; 18 Institute of Microbiology and Infection, School of Biosciences, University of Birmingham, Birmingham, B15 2TT, UK; 19 Advanced Research Computing, University of Birmingham, Birmingham, B15 2TT, UK; 20 MRC-University of Glasgow Centre for Virus Research (CVR), University of Glasgow, Glasgow, UK; 21 Zoonotic Disease Unit, Ministry of Health, Ministry of Agriculture, Livestock and Fisheries, Nairobi, Kenya

**Keywords:** dog-mediated rabies, field sequencing, lyssavirus, MinION, nanopore, neglected tropical diseases, phylogenetic, rabies virus, whole genome sequencing, zoonoses, surveillance

## Abstract

Genomic surveillance is an important aspect of contemporary disease management but has yet to be used routinely to monitor endemic disease transmission and control in low- and middle-income countries. Rabies is an almost invariably fatal viral disease that causes a large public health and economic burden in Asia and Africa, despite being entirely vaccine preventable. With policy efforts now directed towards achieving a global goal of zero dog-mediated human rabies deaths by 2030, establishing effective surveillance tools is critical. Genomic data can provide important and unique insights into rabies spread and persistence that can direct control efforts. However, capacity for genomic research in low- and middle-income countries is held back by limited laboratory infrastructure, cost, supply chains and other logistical challenges. Here we present and validate an end-to-end workflow to facilitate affordable whole genome sequencing for rabies surveillance utilising nanopore technology. We used this workflow in Kenya, Tanzania and the Philippines to generate rabies virus genomes in two to three days, reducing costs to approximately £60 per genome. This is over half the cost of metagenomic sequencing previously conducted for Tanzanian samples, which involved exporting samples to the UK and a three- to six-month lag time. Ongoing optimization of workflows are likely to reduce these costs further. We also present tools to support routine whole genome sequencing and interpretation for genomic surveillance. Moreover, combined with training workshops to empower scientists in-country, we show that local sequencing capacity can be readily established and sustainable, negating the common misperception that cutting-edge genomic research can only be conducted in high resource laboratories. More generally, we argue that the capacity to harness genomic data is a game-changer for endemic disease surveillance and should precipitate a new wave of researchers from low- and middle-income countries.

## Abbreviations

RABV, rabies virus; LMICs, low- and middle-income countries; ONT, Oxford Nanopore Technology; APHA, Animal and Plant Health Agency; RITM, Research Institute for Tropical Medicine; UNITID, University of Nairobi Institute of Tropical and Infectious Diseases; WGS, whole genome sequencing; RIDT, rapid immunodiagnostic test.

## Introduction

Surveillance is critical to inform infectious disease management and control programmes ( Klepac *et al.*, 2013; Molyneux *et al.*, 2004) and genomic data is emerging as a powerful new surveillance tool ( Cotton *et al.*, 2018). Pathogen sequencing has become increasingly routine in well-resourced public health laboratories, but in low- and middle-income countries (LMICs) where most emerging and endemic zoonoses occur, sequencing capacity is more limited and the direct application of genomics-informed surveillance is still at an early stage ( Balloux *et al.*, 2018; Folarin *et al.*, 2014; Gardy & Loman, 2018).

Oxford Nanopore Technologies’ (ONT) MinION machine has revolutionized genomic sequencing capacity ( Johnson *et al.*, 2017). This portable, low-cost sequencer has supported innovative “lab-in-a-suitcase” platforms to diagnose crop pathogens *in situ* ( Boykin *et al.*, 2018; Boykin *et al.*, 2019) and enable real-time outbreak surveillance of emerging pathogens, such as Ebola, Yellow Fever and Zika viruses ( Faria *et al.*, 2018; Faria *et al.*, 2016; Quick *et al.*, 2015; Quick *et al.*, 2016; Quick *et al.*, 2017). There is potential to extend genomic technologies to endemic pathogens such as rabies, foot-and-mouth disease, brucellosis and bovine tuberculosis, which are responsible for major impacts on people’s health and livelihoods on a daily basis ( Cleaveland *et al.*, 2017; Danielsen *et al.*, 2019; Halliday *et al.*, 2015). Such an approach could reduce the lag time from sample to sequence and epidemiological action that is typical for these neglected diseases, while simultaneously building genomic capacity in LMICs. Investing in the surveillance of these endemic pathogens builds core capacities necessary for the detection of unusual disease emergence events that precede epidemics ( Cleaveland *et al.*, 2017; Halliday *et al.*, 2017).

Genomic surveillance has potential applications for management of a range of endemic zoonoses. Here we focus on the development and deployment of genomic surveillance tools to support rabies control and elimination programmes. Rabies is a devastating, invariably fatal zoonotic disease caused by a single stranded negative-sense RNA virus ( Brunker & Mollentze, 2018). Rabies is globally widespread and can infect and be transmitted by all mammal species, but the vast majority of human deaths worldwide result from bites by infected domestic dogs ( WHO, 2018a). Canine rabies elimination is feasible through sustained well-implemented mass dog vaccination programmes ( Abela-Ridder *et al.*, 2016; Lankester *et al.*, 2014; WHO, 2018b). A global initiative to end human deaths from dog-mediated rabies by 2030 is now underway ( WHO, 2018b) and practical, effective surveillance tools are required to support this campaign ( Benavides *et al.*, 2019; Bitek *et al.*, 2019; Broban *et al.*, 2018; Hampson *et al.*, 2016; Mtema *et al.*, 2016).

Genomic surveillance can inform global efforts to eliminate dog-mediated rabies and may be particularly valuable during the endgame, when the disease can circulate for extended periods at low levels ( Klepac *et al.*, 2013). Endemic rabies typically circulates at low levels in dog populations (e.g. 58 to 384 cases per 100,000 dogs in low to high incidence settings in Tanzania, Hampson *et al.,* 2016) and during endgame stages incidence can drop below detectable levels with traditional surveillance, often based on laboratory-based confirmation of submitted suspect cases. Enhanced surveillance including integrated bite case management can increase case detection up to ten-fold ( Suseno *et al.,* 2019) and genetic data from cases detected during this period is critical in determining their role in maintaining circulation and dispersing infection. For example: differentiating dog rabies variants from other wildlife variants ( Lembo *et al.*, 2007); distinguishing ongoing endemic transmission from incursions ( Bourhy *et al.*, 2016; Mollentze *et al.*, 2014b; Zinsstag *et al.*, 2017); and identifying sources of incursions ( Mollentze *et al.*, 2013; Tohma *et al.*, 2016). Furthermore, the capacity for an informed rapid response to newly detected cases will be pivotal for control efforts to succeed ( Henderson, 2011).

Here we establish methods for rapid whole genome sequencing (WGS) of rabies viruses (RABV) using the MinION sequencer, including a workflow from sample to sequence and the associated bioinformatics pipeline. We also incorporate procedures for downstream analysis, interpretation, and management of genomic data in a surveillance context. Through testing and replication of methods across different locations in Kenya, Tanzania and the Philippines we demonstrate the feasibility of real-time sequencing of RABV to rapidly inform policy decisions and disease management. We further highlight the potential for cost savings that could make routine genomic surveillance of rabies affordable in low-resource endemic settings.

## Methods

**Figure 1.**
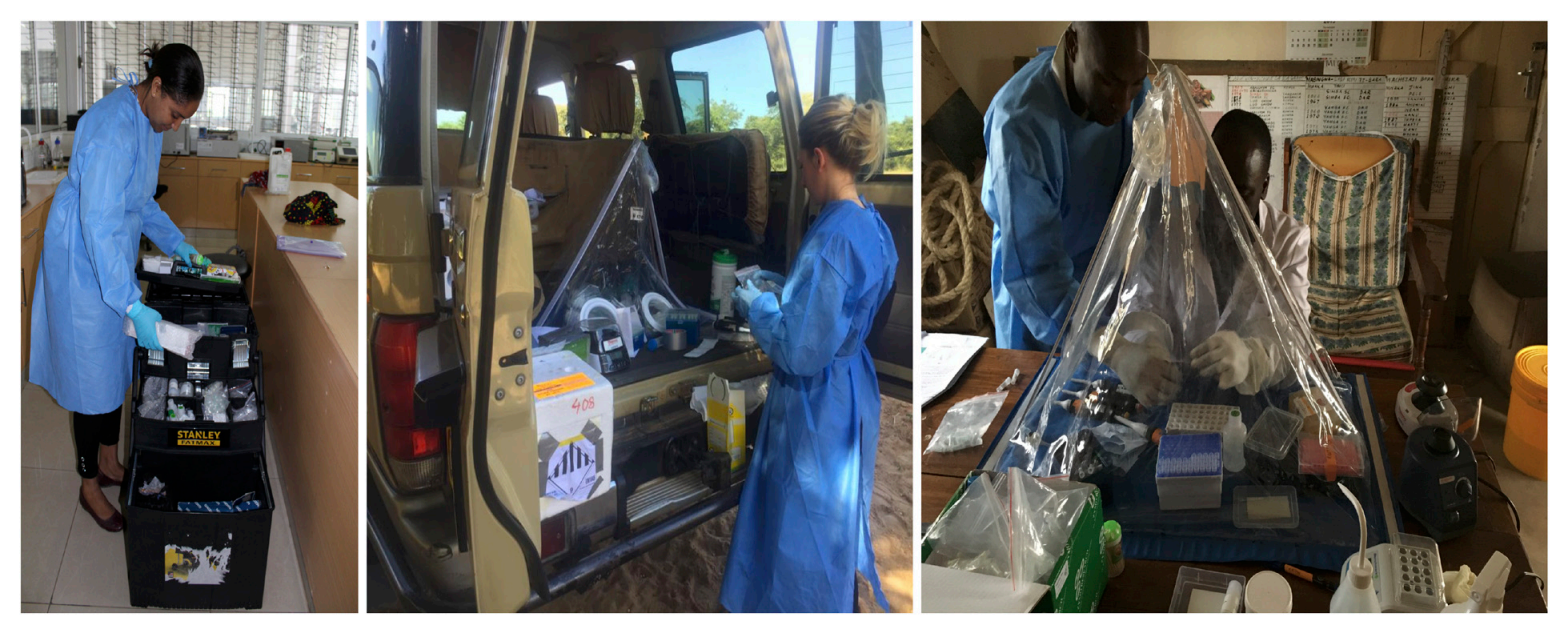
Rabies “lab-in-a-suitcase” setup for sequencing in low-resource settings. **A**) Lab-in-a-suitcase being used at the Nelson Mandela African Institute of Science and Technology, Tanzania; **B**) RNA extractions performed in the back of a vehicle using a portable glove box and battery-powered centrifuge in Makueni District, Kenya; **C**) Laboratory setup for sample inactivations in a district livestock office in Mugumu, Serengeti District, Tanzania.

Rabies positive samples were processed under a range of conditions to generate near whole genome sequences for viral characterization and phylogenetic analysis. Here we describe the sequencing and bioinformatics pipelines and validation process, detailing adaptations required for different settings. Our aim was to establish a protocol that could be used under minimal laboratory infrastructure; therefore, the approach is supported by a lab-in-a-suitcase platform: a portable toolkit to support the steps required to get from sample to sequence ( Figure 1). A comprehensive, up-to-date sample-to-sequence laboratory protocol is available on protocols.io and supporting pipelines can be found on GitHub will be updated with improvements as methods develop. The ARTIC network, a Wellcome funded project to develop the application of genomic surveillance for viral outbreak response, provides comprehensive open-source resources for laboratory and sequencing work, bioinformatics, phylogenetics and analysis. We take advantage of these resources for our rabies workflow. They can be found here on the ARTIC network website and are published in Quick *et al.*, 2017. We also utilise RABV-GLUE ( Singer *et al.*, 2018), a general-purpose resource for analysis, interpretation, and management of RABV genome data, to organise and interpret RABV consensus sequences.

### Samples

**Figure 2.**
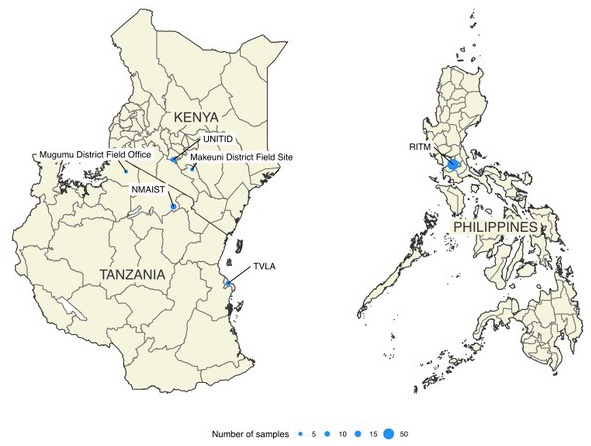
Locations in East Africa where sequencing of rabies viruses was performed. Circles are scaled according to numbers of samples sequenced. Details of laboratory capacity at each site are provided as underlying data ( Brunker, 2019b).

Brain tissue samples were collected by field officers and veterinarians from suspect rabid animals as part of rabies surveillance and research projects across Kenya (n = 9), Tanzania (n = 26, with 2 archived samples processed in the UK) and the Philippines (n = 52). These samples were accessible through existing collaborations and time allowed for their processing whilst optimizing our protocols and delivering training workshops, therefore numbers do not necessarily represent the surveillance capacity in each locality. Samples were obtained predominantly from domestic dogs but also included livestock and wildlife samples; details are provided as underlying data ( Brunker, 2019b). Samples were processed in settings with varying infrastructure and resources, from central diagnostic laboratories to field sites. Details of each site can be found in Table 1 and Figure 2. Rapid immunodiagnostic tests (RIDT), manufactured by BioNote, Inc. (RG18.01, Gyeongi-do, Republic of Korea), were used to confirm rabies positive cases in the field in Tanzania. These kits have been validated as an effective rabies field diagnostic tool ( Léchenne *et al.*, 2016). In Kenya, samples were confirmed positive by the Kenya Medical Research Institute (KEMRI) using the direct rapid immunohistochemical test (dRIT) and tested by RIDT prior to sequencing. In the Philippines samples were tested by the Research Institute for Tropical Medicine (RITM) using the fluorescent antibody test (FAT) ( Abela-Ridder, 2018).

**Table 1.** Locations across Kenya, Tanzania, the Philippines and UK where rabies virus sequencing was conducted. The number of samples sequenced, number of MinION sequencing runs and resources available at each location are detailed. (*six out of the 11 samples were repeats of FIELD2 samples).

Site	Abbreviation	Location (City, Country)	# Samples sequenced (# MinION runs)	Year	Classification	Resources					
						Containment level 2	Freezer -80°C	Freezer -20°C	Laboratory	Back-up Generator	Standard lab equipment ^1^
Tanzania Veterinary Laboratories Agency	TVLA	Dar es Salaam, Tanzania	9 (2)	2017	Research/ diagnostic lab	**✓**	**✓**	**✓**	**✓**	**✓**	**✓**
Research Institute for Tropical Medicine	RITM	Manilla, Philippines	52 (3)	2019	Research/ diagnostic lab	**✓**	**✓**	**✓**	**✓**	**✓**	**✓**
Mugumu District field office	FIELD1	Mugumu, Tanzania	4 (2)	2017	Field site			**✓**			
University of Nairobi Institute of Tropical and Infectious Diseases	UNITID	Nairobi, Kenya	11* (1)	2019	Research laboratory	**✓**	**✓**	**✓**	**✓**	**✓**	
Makeuni Field site	FIELD2	Makueni District, Kenya	4 (1)	2019	Field site (RNA extractions only)						
Institute of Biodiversity, Animal Health & Comparative Medicine, University of Glasgow	IBAHCM	Glasgow, UK	1 (1)	2017	Research laboratory (from RNA stage)		**✓**	**✓**	**✓**	**✓**	**✓**
Animal & Plant Health Agency	APHA	Surrey, UK	1 (1)	2016	Research/ diagnostic lab	**✓** CL3	**✓**	**✓**	**✓**	**✓**	**✓**
Nelson Mandela Institute of Biodiversity, Animal Health & Comparative Medicine	NMAIST	Arusha, Tanzania	12 (3)	2017, 2018	Research laboratory	**✓**	**✓**	**✓**	**✓**		**✓**

[1] Centrifuge and thermocycler available for use

### Equipment

A full suite of portable, battery-powered equipment and laboratory consumables was put together based on the ARTIC network’s published kit list. Reagents that required cold chain transportation were carried in polystyrene temperature control boxes packed with ice packs frozen at -20 or -80°C prior to use. This equipment, including reagent boxes, could be packed into standard airline sized checked luggage (lab-in-a-suitcase) for transportation ( Figure 1).

### Sample preparation and RNA extraction

Methods to perform sample processing and RNA extraction varied according to resources available on site. In research or diagnostic laboratories, samples were handled in containment level (CL) 2+ or CL3 conditions until sample inactivation. At field sites, samples were handled in a portable glove box (UY-33666-50, Cole Parmer). RNA extraction was performed using the Zymo Quick RNA Miniprep kit (R1054, Zymo Research) according to the manufacturer’s protocol. Sample preparation varied according to the state of the sample (fresh/frozen, homogenised/non-homogenised) and its preservation (RNAlater [Invitrogen], DNA/RNA Shield [Zymo Research], 50% glycerol-saline solution). Recommended methods for different sample types can be found in the manufacturer’s instructions but generally the following protocol was used. A small amount of brain tissue (50–100mg) was transferred from the original sample tube, using a wooden applicator stick or toothpick, to a 2ml reinforced homogeniser tube containing 1.4mm ceramic beads (Qiagen) and ~1ml of Zymo kit RNA lysis buffer. The sample was homogenised using a Terralyzer (Zymo Research) with 30 second pulses until fully homogenised and left to inactivate for 2 minutes. When a Terralyzer was unavailable, samples were manually homogenised using a toothpick. Lysed samples were processed according to manufacturer’s column-based RNA purification protocol with in-column DNase I treatment to remove genomic DNA. In brief, lysed samples were added to filter columns and subject to a series of centrifugation steps to selectively bind RNA and purify (ethanol washes) and finally eluted. Purified viral RNA was eluted in 50µl DNase/RNase-Free Water. Aliquots of four samples stored in RNA later were shipped on dry ice from Tanzania to the Animal & Plant Health Agency (APHA, Weybridge UK) in 2017. These, and two samples used for validation (see below), were extracted according to a previously described Trizol-based method at APHA ( Brunker *et al.*, 2015). Briefly, 50–100mg of brain tissue was homogenised in 1ml Trizol and 200µl chloroform added to enable phase separation. RNA separates into the upper aqueous phase, which is removed and RNA precipitated using isopropanol. This is followed by centrifugation to pellet RNA and ethanol washes to purify, with elution in DNase/RNase-Free Water.

### Multiplex primer design and validation

Multiplex primer schemes were designed for RABV using Primal Scheme. For Tanzania, a set of 10 reference sequences from datasets curated and filtered in RABV-GLUE were chosen to represent the known RABV diversity in Tanzania (in file order submitted to Primal Scheme: KR906748.1, KR906747.1, KY210263.1, KY210264.1, KY210227.1, KR906774.1, KR906763.1, KR906742.1, KR534218.2, KY210305.1). Settings were applied to generate primers for 400bp amplicons across the entire genome (~11923bp), with an overlap of 50bp. A short amplicon length was chosen due to previous experience of poor sample quality from field collected and stored samples resulting in RNA fragmentation and a low viral copy number ( Brunker *et al.*, 2015; Brunker *et al.*, 2018). The first scheme created, rabvTanzDg, was tested on archived Tanzanian RABV RNA samples at APHA following the protocol described in Quick *et al.* (2016). Two sequencing validations were performed: 1) Amplicons from one sample, RV3227, were sequenced on an Illumina NextSeq to confirm the amplification of individual products across the genome. This sample had already been sequenced using a PCR-free metagenomic approach, briefly described in section “Additional Sequencing” (described in Brunker *et al.*, 2015), providing a comparison between library preparations; 2) Subsequently, amplicons from another sample (RAB16025) were sequenced on both the MinION and NextSeq (fragmented before sequencing on NextSeq). In addition, metagenomic sequencing was conducted on the NextSeq for this sample. A second RABV scheme was produced for the Philippines (rabvSEasia) following the same approach and used directly for MinION sequencing (reference sequences: KX148260, KX148263, N-gene 99% consensus sequence of [ AB116581, AB116582, AB683592: AB683635], AB981664, KX148255, JN786878, KX148250, KX148254, EU293111, KX148248, KX148266).

### DNA preparation

Protocols and reagents varied slightly due to availability and as protocols evolved; the latest version of the protocol is available on the GitHub resource and Figshare ( Brunker, 2019d). Initially, extracted RNA was reverse transcribed using Protoscript II First Strand Sequencing kit (E6560, NEB) under manufacturer’s instructions. At later stages this was changed to Lunascript RT Supermix (E3010, NEB) in a reduced reaction volume of 10μl (2μl Lunascript + 3μl nuclease-free water +5μl RNA). Multiplex PCR was conducted with 2 to 2.5 µL of cDNA, the RABV primer scheme and Q5 High Fidelity Hot-Start DNA Polymerase (M0493, NEB) following the two-step PCR protocol ( Quick *et al.*, 2017), with the following PCR cycling conditions: 98°C heat inactivation for 30sec, followed by 30–40 cycles of denaturation at 98°C for 15sec and a combined extension/annealing step of 65°C for 5min. PCR products were purified using a 1x solid-phase reversible immobilization bead (SPRI) cleanup (Ampure XP, Beckman-Coulter or PCRClean DX,Aline Biosciences) and concentrations measured using a Qubit dsDNA High Sensitivity kit (Q32851, Thermofisher) on a Qubit 3.0 fluorimeter (ThermoFisher). In field sites, or when standard thermocyclers in laboratories were unavailable, PCR was performed using a portable MiniPCR machine (Amplyus). This was powered by a lithium battery (PowerAdd Pilot Pro2 85Wh LiION 9-20V) for the full PCR cycle when power was intermittent or unavailable. A negative control (water) was included at the cDNA synthesis stage for each batch of samples and as part of the sequencing library. Some of the first runs in Tanzania did not include a negative control as part of the sequencing library as the protocol was still being finalised. However, a negative control up to the PCR stage was always included as part of sample preparation. If the negative control was >1ng/μl by Qubit quantification, sample preparation was repeated from RNA extraction.

### MinION library preparation and sequencing

Library preparation for the MinION was conducted using Ligation Sequencing 1D (SQK-LSK108/SQK-LSK109, ONT) and Native Barcoding kits (EXP-NBD103/EXP-NBD104/EXP-NBD114, ONT) on FLO-MIN106 (R9.4/R9.4.1, ONT) flowcells according to the manufacturer’s instructions and modifications as per Quick *et al.* (2017). Briefly, DNA prepared in the previous step (PCR amplicon pools for each sample were pooled) was end-repaired and dA-tailed using an UltraII End Prep Reaction Module (E7442, NEB) followed by ligation of barcodes using the NEBNext UltraII Ligation module (E7595, NEB) in a “one-pot” reaction. Following barcode ligation, samples were purified using a SPRI bead cleanup and pooled together into a single tube before ONT adaptor ligation with the NEBNext UltraII Ligation module. Ligated DNA was then cleaned using a SPRI bead clean-up with ABB/SFB (SQK-LSK108/SQK-LSK109, ONT) washes and final library elution in ONT’s elution buffer (SQK-LSK108/SQK-LSK109, ONT). With the latest kit versions, up to 24 native barcodes are available, but multiplexing varied by run according to available samples and barcodes, with multiplexed MinION runs of three to 24 samples conducted. Sequencing libraries were prepared with a negative control (non-template control from cDNA synthesis), which was sequenced as a barcoded sample. Sequencing was performed on a Dell Latitude E5470 laptop (CPU: 6th Generation Intel Core i7-6820HQ [Quad Core, 2.7GHz, 8MB cache]; Memory: 16GB RAM; Storage: 1TB solid state drive) using MinKNOW (version available at the time, v1.7.3 to 18.12.9) with live basecalling turned off. The MinION functions as a “read-until” platform, whereby the length of sequencing runs is determined by the user when sufficient data has been produced (maximum 48h); therefore, this varied between runs. An offline version of MinKNOW provided by ONT was used due to varying internet quality at different locations. To ease the installation and use of bioinformatic pipelines in the field, a lab-on-an-SSD approach was used to ensure a working bioinformatic environment was always available. The lab-on-an-SSD is a bootable USB3 drive containing a linux installation, ONT software and bioinformatic analysis software, available from the ARTIC network resources, that offers a plug-and-play bioinformatic environment for most laptops. SSD image available as an open-source resource here: https://github.com/artic-network/fieldbioinformatics/tree/master/lab-on-an-ssd),

### Additional sequencing

Four samples shipped to the UK and extracted at APHA were sequenced using the metagenomic approach previously described ( Brunker *et al.*, 2015). Briefly, this involved host genomic DNA depletion of extracted total RNA using the on-column DNase treatment in RNeasy plus mini kit (74134, Qiagen) followed by first- and second-strand cDNA synthesis with a cDNA synthesis system kit (04379012001, Roche) and library preparation with a Nextera XT sample preparation kit (FC-131-1024, Illumina).

### Cost estimates

**Figure 3.**
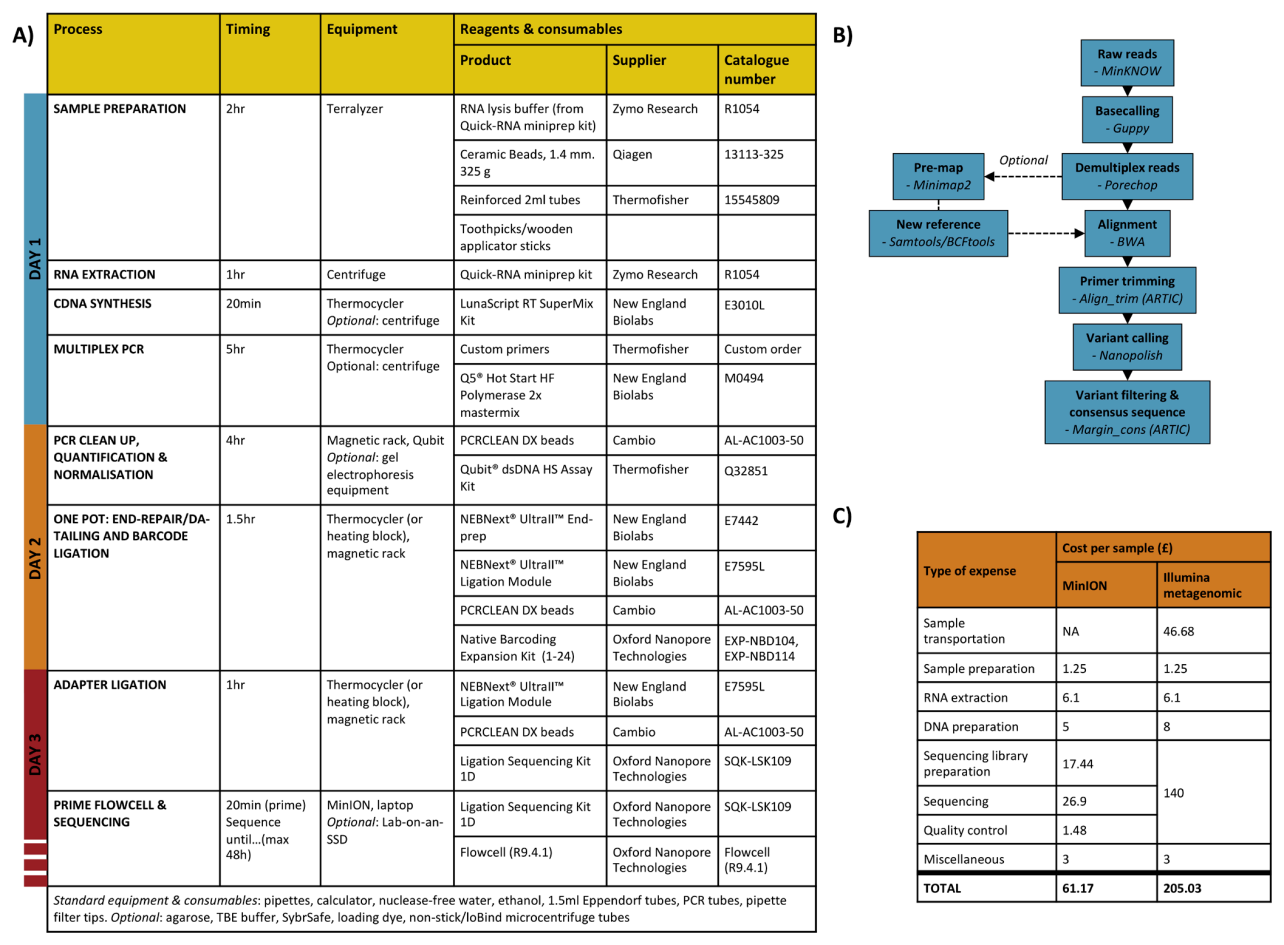
Workflow from sample to sequence. **A**) Table of laboratory workflow steps with estimated timings and details of important reagents/consumables; **B**) Bioinformatics workflow diagram; **C**) Estimated cost per sample for end-to-end rabies virus sequencing in-country using a PCR-based MinION approach versus the PCR-free metagenomic approach previously used in the UK. Costs assume a bulk buy of reagents for 100 reactions and a multiplex capacity of 24 samples per flowcell for MinION sequencing.

The costs per sequenced sample were estimated for the MinION-based multiplex PCR approach and the previously used Illumina-based metagenomic approach ( Brunker *et al.*, 2015; Brunker *et al.*, 2018). These estimates include processing from sample to sequence, i.e. incorporating all costs from the workflow shown in Figure 3 and any transportation costs associated with the shipment of samples to laboratory facilities. Reagents and consumable costings assume a bulk buy for at least 100 samples. For metagenomic sequencing, costs were based on previous quotes from UK sequencing facilities to process RABV samples, including sample shipment from Tanzania to the UK.

### Bioinformatics and data management

***i) Consensus sequence generation.*** Bioinformatic pipelines were run on the same laptop used for sequencing. A RABV version of the ARTIC network open-source bioinformatics pipeline ( Quick *et al.*, 2017) was used to generate consensus sequences for each barcoded sample ( Figure 3B). Briefly, raw files were basecalled with the latest ONT software at the time (Albacore or Guppy) and demultiplexed with Porechop using stringent settings to select reads with barcodes at both ends of the read. Demultiplexed reads were then mapped with BWA ( Li & Durbin, 2009) to a reference genome chosen according to the country of origin (Tanzania/Kenya: Genbank accession no. KF155002; Philippines: Genbank accession no. KX148260). Prior to variant calling, primer sequences were trimmed using their genome coordinates and their genome coverage normalised. Variants were called using Nanopolish ( Loman *et al.*, 2015), which uses MinION signal-level data to “polish” the genome assembly. Consensus sequences were generated under the condition that non-overlapping primer binding sites and sites for which coverage was <20X were masked by ambiguity code N. Illumina reads were processed according to a previously described method ( Brunker *et al.*, 2015). Briefly, Genome Analysis Toolkit ( Poplin *et al.,* 2017) was used to identify high quality SNPs using filters on strand bias (FS>60, SOR>4), mapping quality (MQ<40, MQRankSum≤12.5), read position (ReadPosRankSum≤8), and depth of coverage (DP<5). Consensus sequences were produced with a 75% majority basecall rule. Raw and processed sequence data files were stored locally on the laptop’s internal hard drive or external SSDs. Data was transferred to University of Glasgow servers for storage and backup on return to the UK.

***ii) Analysis/interpretation.*** Major and minor clade assignments were obtained for newly generated RABV sequences using Maximum Likelihood Clade Assignment (MLCA) as implemented in RABV-GLUE ( Singer *et al.*, 2018). MCLA uses RAxML's Evolutionary Placement Algorithm ( Stamatakis, 2014) to select high-likelihood branch placements for a query virus sequence in a previously calculated phylogeny of virus reference strains. The reference phylogeny in RABV-GLUE was reconstructed using maximum likelihood, and contains a representative set of RABV sequences obtained from recent studies of RABV diversity ( Kuzmin *et al.*, 2012; Troupin *et al.*, 2016). Following MLCA, RABV-GLUE was used to import relevant sequences from GenBank to compare to the new sequence data from each area i.e. East Africa and the Philippines. GenBank sequences were curated from the same minor clade associated with each area according to MLCA and both GenBank and MinION sequences were filtered to include only sequences with >90% genome coverage. For sequences produced for this study that was 24 out of 34 sequences from East Africa and 44 out of 52 sequences from Philippines. Alignments were produced using MAFFT ( Katoh & Standley, 2013) implemented in RABV-GLUE. Maximum likelihood phylogenies were produced for each of the alignment sets using RAxML implemented in RABV-GLUE with a General Time Reversible model of nucleotide substitution with the gamma model of rate heterogeneity and a proportion of invariant sites and 1000 bootstraps. Outgroups consisting of RABV genomes from other minor clades were included in tree building.

### Training in MinION sequencing

Workshops were conducted in Kenya (11 participants) and the Philippines (10 participants) to provide hands-on training in MinION library preparation, sequencing and basic bioinformatics. Participants typically had a background in molecular biology but most had never prepared sequencing libraries. RABV RNA from local archived samples was used for sequencing following the sample-to-sequence method described above. Basic bioinformatic training included an introduction to the command line and use of the lab-on-an-SSD resource with the RABV version of the pipeline. In addition to workshops, training of local researchers was conducted on a one-to-one or small group basis at every opportunity. Consent from participants was requested as part of a questionnaire to give feedback on training workshops. Only those that consented provided feedback (75% of participants). Given that no identifying or sensitive information was collected, no ethical approval was sought for this questionnaire.

## Results

### Validation of pipeline in UK laboratory

**Figure 4.**
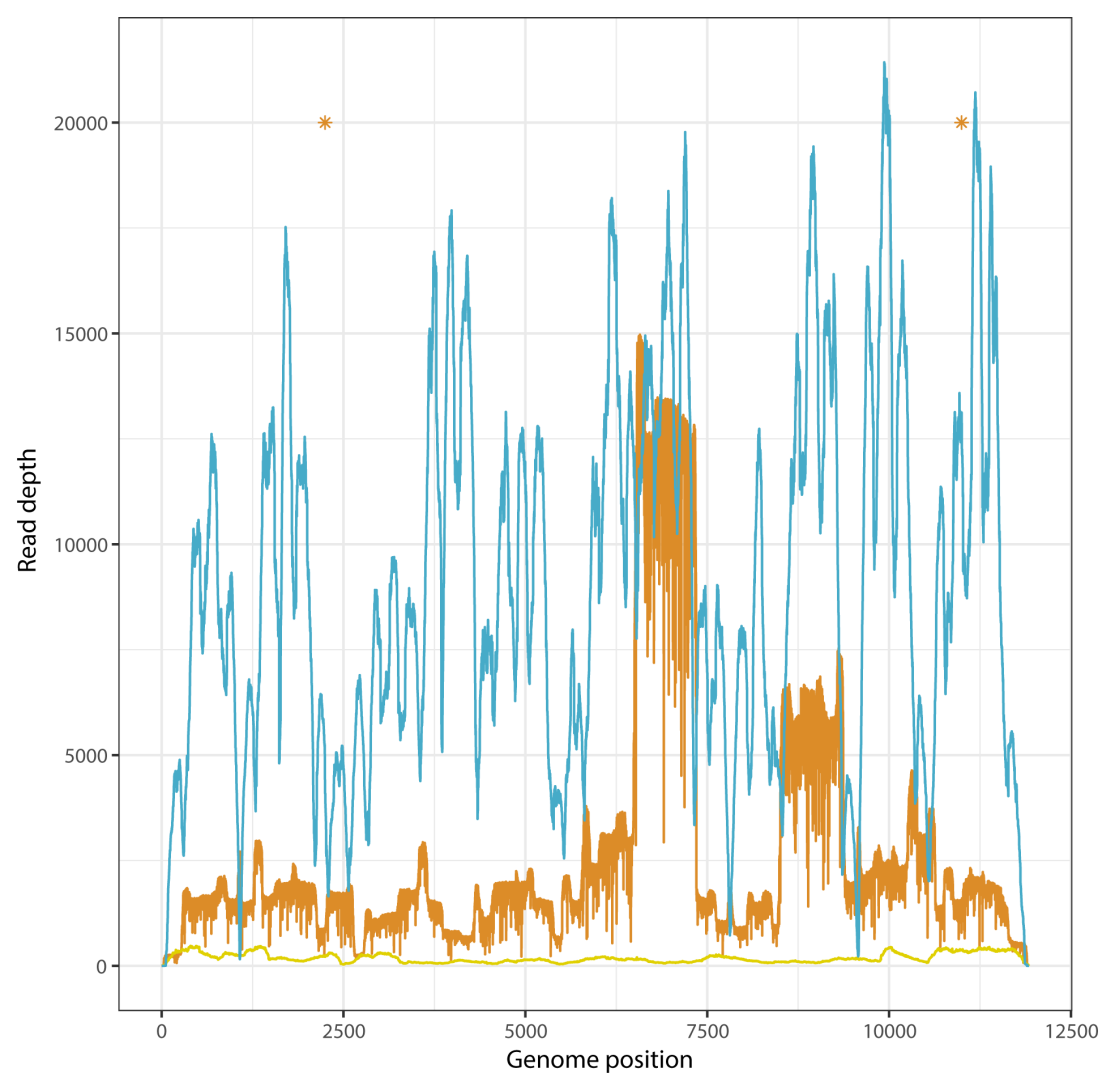
Genome coverage profiles for a Tanzanian dog rabies virus sample sequenced by different methods. Blue: PCR amplicons (fragmented by Nextera library preparation) sequenced on Illumina NextSeq; orange: PCR amplicons sequenced on MinION sequencer; yellow: metagenomic sequencing on Illumina NextSeq. Asterisks indicate locations of single nucleotide polymorphisms between Illumina and MinION consensus sequences.

Amplicons from sample RV3227 sequenced on the NextSeq platform produced over 22million mapped reads and an average read depth of 6971 with >99% genome coverage. The same sample sequenced using the PCR-free metagenomic approach on the NextSeq produced 9498 mapped reads (0.4% of the total reads sequenced) with >99% genome coverage. Consensus sequences from each approach were identical. Amplicons from sample RAB16025 were sequenced on both NextSeq and MinION platforms, and metagenomic sequencing was also performed. Comparison between the three consensus sequences revealed 100% identity between Illumina consensus sequences (metagenomic vs PCR) and 99.98% identity between Illumina and MinION sequences. There were two SNPs between Illumina and MinION consensus sequences, highlighted in Figure 4.

### In-country genome sequencing

RABV samples were sequenced across a range of settings in Kenya, Tanzania and the Philippines between 2017–2019 ( Table 1 and Figure 2). Using a lab-in-a-suitcase approach provided the flexibility to ensure that the necessary resources were available for sequencing in resource-limited settings but also in well-resourced laboratories if particular equipment was not available. In total, 84 brain tissue samples and one salivary gland sample were processed from sample to sequence in their respective country of origin. For all samples, a consensus was generated for all sites across the genome where coverage was ≥20x ( Brunker, 2019a). The majority of samples (~80%) achieved >90% genome coverage at a depth of at least 20x ( Brunker, 2019b). Due to practical constraints, sequencing run times varied; therefore, the total throughput from each run was different. Instances where coverage dropped below 20x may have been improved with longer sequencing times.

The total processing time from sample to sequence-ready library for a batch of 24 samples was ~15 hours ( Figure 3A). Sequencing run length varied batch to batch and was determined by practical constraints and the expected output required for 100x read coverage for *n* samples in a run (and assuming loss of reads during demultiplexing). Typically, we found 5–8 hours was sufficient to get adequate coverage for multiplexed runs. For most samples, the time taken to process them end-to-end was 2–3 days when accounting for reasonable working hours (which are often restricted in laboratories) and processing a large number of samples (up to 24) for each run. The most time-consuming parts of the protocol were sample preparation for RNA extraction due to variation in the quality of brain tissue; PCR setup for a large number of samples (24 samples = 48 PCR reactions); and the normalisation of PCR products for sequencing libraries - samples with high concentrations of DNA often required an additional dilution step to provide suitable volumes (>1μl) for pooling after normalisation ( Figure 3). Workflow times can be reduced with multiple workers (estimated timings assume only one person is doing the work) and good preparation e.g. prior tube labelling/setup, the preparation of next steps during incubations, preparation of stock solutions ahead of time etc, but careful work is required to follow workflow steps to avoid mistakes and introducing contamination.

Negative controls were included in each batch of samples processed and sequenced in most runs. Contamination was found in some batches, particularly during group training exercises (e.g. Philippines workshop batch), highlighting the importance of this quality control step. When contamination was detected during PCR quantification steps (concentration >1ng/μl), sample preparation was repeated from RNA extraction. However, some cross-contamination was not apparent until sequencing; therefore, a negative control should always be included in the sequencing library. A low level of cross-contamination was noted in all sequencing runs (a few hundred reads), often caused by amplicons with particularly high levels of amplification.

### Costings

We estimated the full cost of sample-to-sequence preparation for RABV samples to give a better understanding of the costs involved to process field collected samples ( Brunker, 2019c). The cost reduced as improvements to the protocol and multiplexing capacity were made. Early runs, which had a low number of samples per flowcell and were used mainly for validation, were approximately £160 per sample. This reduced to ~£60 per sample at full multiplexing capacity, i.e. 24 samples per flowcell ( Figure 3C). Furthermore, the development of an effective washing procedure for flowcell reuse (“Nuclease-flush”, Oxford Nanopore) means that, potentially, multiple runs per flowcell could be achieved. Therefore, we anticipate that this will reduce even further to ~£35 to £45 per sample (based on 2–6 reuses of one MinION flowcell). For example, a similar PCR-based sequencing approach for Zika virus in Brazil was able to yield ~150 samples per flowcell by making use of the nuclease-flush between runs (S. Hill, University of Oxford, personal communication). In comparison, sample shipment to the UK followed by sample-to-sequence costs for an Illumina-based metagenomic approach used previously for RABV WGS costs an estimated ~£205 per sample ( Figure 3C). In terms of bioinformatic/data management costs, the SSDs used were typically purchased for £75–80 but are an optional component of the workflow and therefore not included in the overall estimate.

### Training

21 participants from veterinary and public health laboratories across Tanzania, Kenya and the Philippines took part in four-day sequencing workshops held in-country. Each participant prepared one sample during group laboratory sessions, providing hands-on experience of end-to-end preparation for MinION sequencing. Participants typically had experience in molecular laboratory methods such as PCR but most had never prepared sequencing libraries before. Feedback questionnaires revealed that participants were overall very satisfied with training workshops but indicated a desire for more comprehensive bioinformatics training. At the end of each workshop, participants were asked to submit a mini research proposal and a commitment from their research institute to support MinION genomic work in order to obtain a MinION starter pack and additional reagents. In total, four MinION starter kits were provided to groups of researchers from the workshops to kick-start the application of their new training skills for genomics research. At the time of writing none of the groups had conducted sequencing mostly due to problems procuring additional reagents (either due to funding or supplier issues). However, we have offered ongoing support e.g. provision of extra reagents, linking up with local expertise to ensure projects can be completed and all are expected to sequence within the next few months.

### Consensus sequence analysis

**Figure 5.**
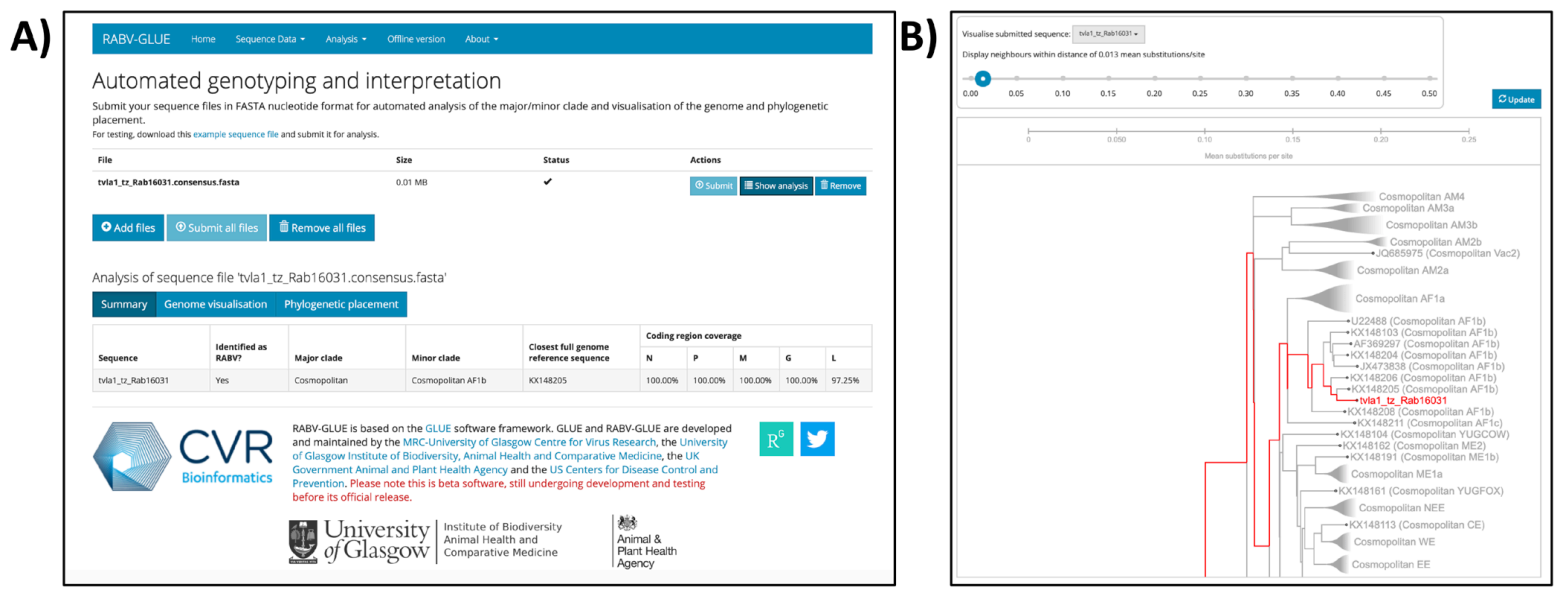
Example output from the online RABV-GLUE: an online tool for automated genotyping and interpretation of rabies virus (RABV) sequence data. **A**) Major and minor clade assignments for a Tanzanian RABV whole genome sequence (sample id=Rab16031, GenBank accession=MN726824); **B**) Phylogenetic placement of the sequence within the RABV-GLUE rabies virus reference phylogeny. This figure has been reproduced with permission from the University of Glasgow.

**Figure 6.**
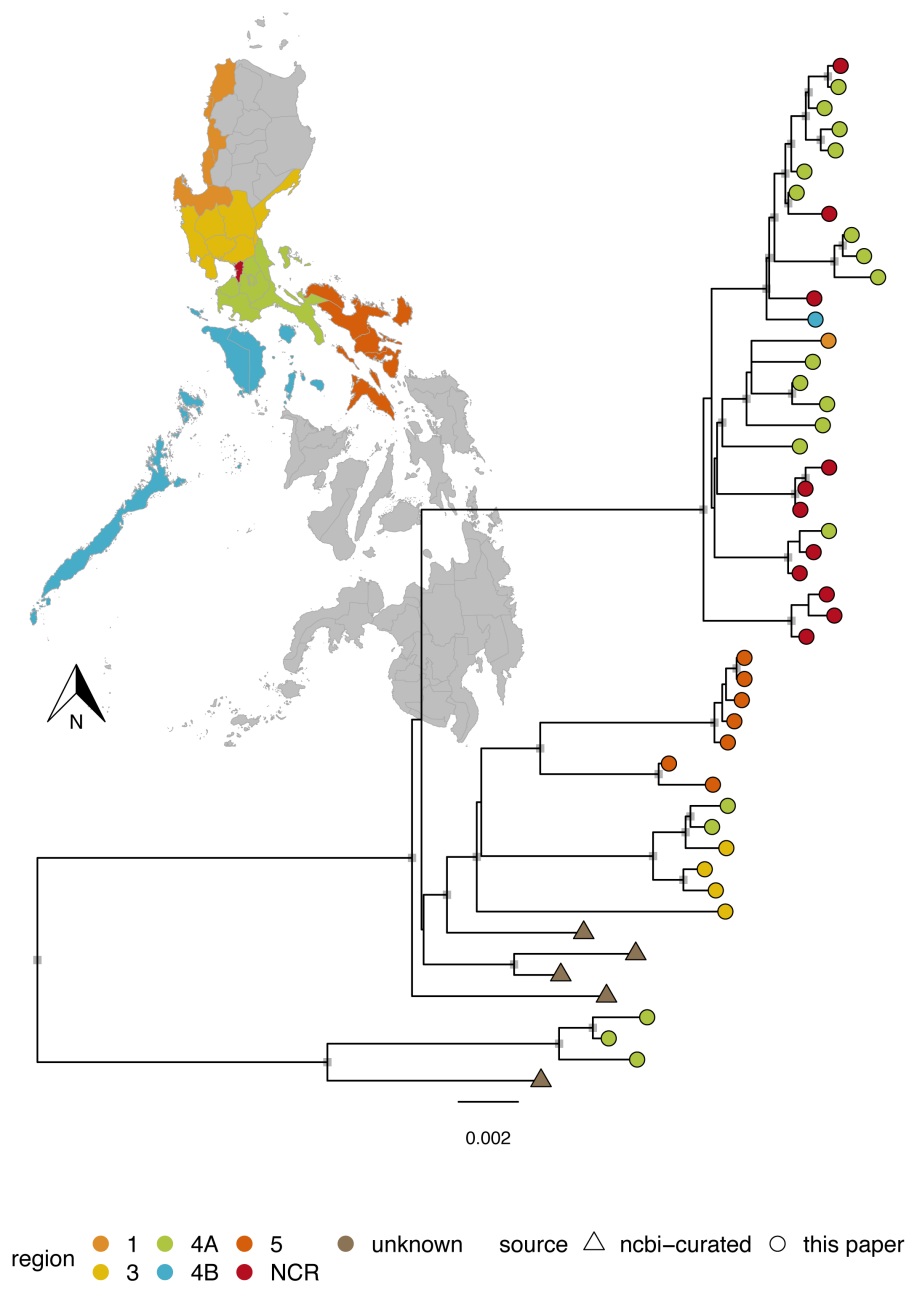
Maximum likelihood tree of rabies virus genomes from the Philippines. Genomes from the rabies virus Asian minor clade SEA4, which were sequenced in this study (n=44) and curated from GenBank (n=5). Colours indicate the administrative region associated with each sequence as shown on the map and internal node symbols indicate bootstrap support ≥80. Genbank sequences GU358653 and GU647092 representing Asian minor clades SEA2a and SEA2b were used as an outgroup (branch not shown). Administrative shapefiles were obtained from https://www.diva-gis.org/datadown and plotted in R.

**Figure 7.**
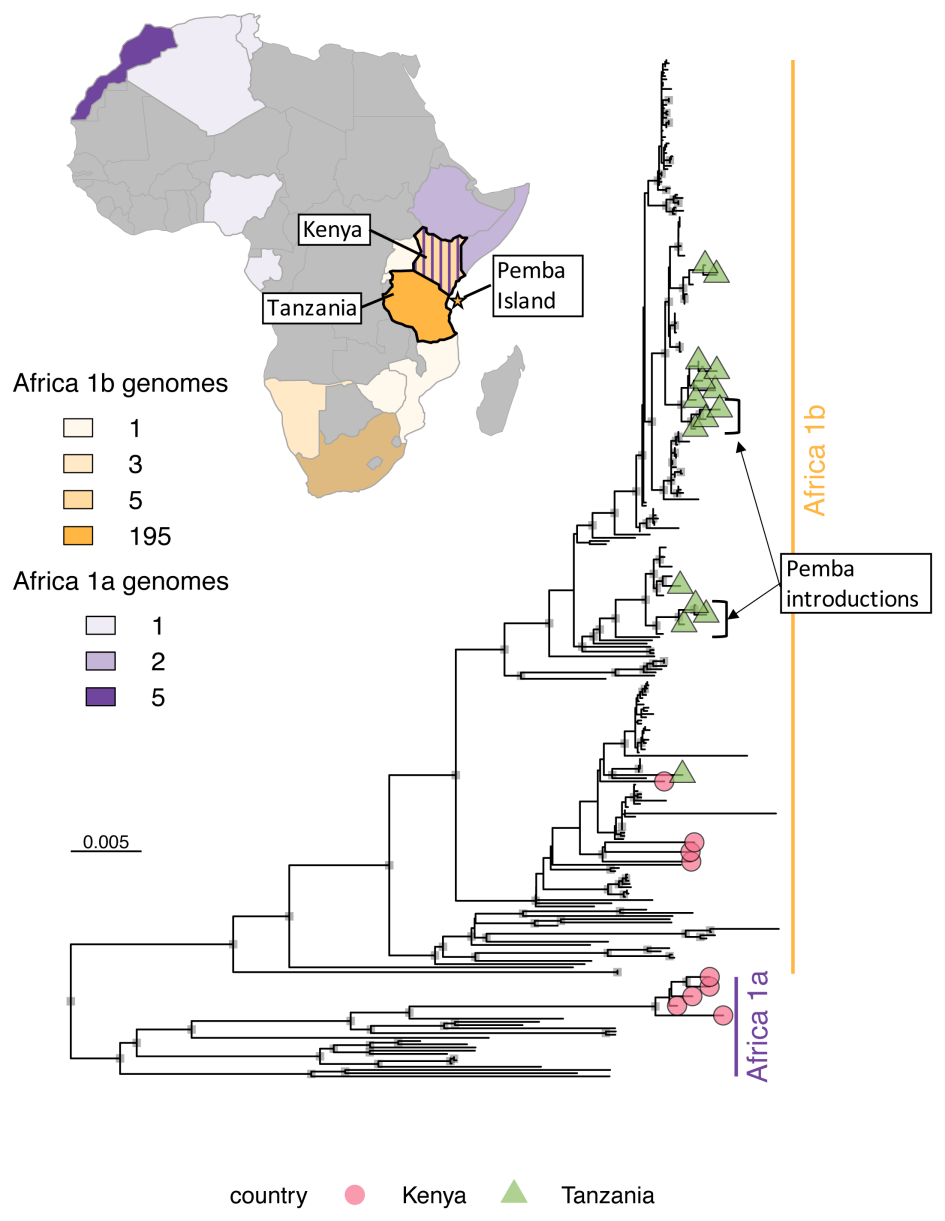
Maximum likelihood tree of rabies virus genomes from Kenya and Tanzania. Genomes belonging to two minor clades in the rabies virus Cosmopolitan clade are highlighted, Africa 1a and Africa 1b, which were sequenced in this study (tip shapes, n=24) and curated from GenBank (n=220). The country of origin and number of sequences in each minor clade are annotated on a map of Africa and the location of Pemba sequences from a 2016/17 outbreak is annotated on the tree, indicating multiple introductions from the mainland. Genbank sequences representing other minor clades ME1a, ME1b and ME2 (KX148162, KX148190, KX148191) were used as an outgroup (branch not shown). Maps were generated with package rWorldMaps in R.

Major and minor clade assignments were obtained for all sequences using RABV-GLUE (see example in Figure 5), even for sequences with very poor consensus genome coverage of coding regions (as little as 15.1%; see underlying data; Brunker, 2019b). Philippines sequences were all identified as Asian SEA4, a minor clade so far detected exclusively in the Philippines ( Saito *et al.*, 2013; Tohma *et al.*, 2016, source: RABV-GLUE). Subdivision within the Philippines subclade indicates regional phylogeographic structure, as found previously ( Saito *et al.*, 2013; Tohma *et al.*, 2016) but also further subdivision that may indicate structure at a finer spatial resolution ( Figure 6). Tanzanian sequences were all from the Africa 1b lineage, as found previously ( Brunker *et al.*, 2015; Brunker *et al.*, 2018). Interestingly, we detected two minor RABV subclades in Kenya; Africa 1a and Africa 1b ( Figure 7). The limited RABV genetic data available for Kenya prior to our work (one WGS, nine complete/partial nucleoprotein sequences) are all Africa 1b subclade. 

## Discussion

Through a series of sequencing applications and trainings conducted in different settings between 2017–2019, we demonstrate the feasibility of a cost-effective, in-country pipeline for WGS of RABV using the MinION. Although challenges exist, we show they are not insurmountable. Our pipeline offers both a scalable, low cost workflow that can be particularly beneficial for sequencing a large set of archived samples and a rapid sample-to-sequence turnaround for priority samples to inform decision-making for disease management. We share our protocols and guidance to facilitate WGS of RABV, even in resource-limited settings, discuss its limitations and prospects for ongoing application.

Genomic data has become an important aspect of contemporary infectious disease surveillance ( Gardy & Loman, 2018). Yet for rabies and other neglected endemic diseases it is underused - largely limited to academic studies and retrospective analyses with more limited actionable outputs. Even well-funded research projects can take up to six months from sample collection to dissemination of results (e.g. Brunker *et al.*, 2018; Boykin *et al*., 2019), impeding the quick decision-making necessary for timely disease control. Using our MinION-based sequencing protocol, RABV samples can be sequenced in-country in a matter of days and at a cost much lower than third-party sequencing facilities ( Figure 3C), which typically requires the export of samples. We envision two broad applications of our protocols, for: 1) cost-effective batch sequencing of archived samples to characterize circulating RABV diversity, and 2) rapid *in situ* genomic surveillance of new cases, particularly during the endgame when such information can target appropriate responses. Many countries have repositories of archived tissue samples from rabid animals, which could be sequenced in bulk and therefore at low cost. These samples are potentially very valuable to characterise historical RABV diversity in a locality from which to compare to contemporary sequences. This comparison can show whether lineages continue to circulate or have disappeared, perhaps as a result of rabies control efforts. The latter option of rapid *in-situ* sequencing makes less use of cost-cutting measures such as multiplex sequencing, but the benefit of genomic surveillance to gain time-critical insights into persisting or re-emerging RABV far outweigh the expense of re-emergence. Further options to reduce costs when sample numbers are low include combining runs with other pathogens or using ONT’s low-cost, lower throughput Flongle flowcells (e.g. Grädel *et al.,* 2019).

To facilitate replicable sequencing in different settings, we provide detailed instructions for each stage of sample processing. Each stage independently is relatively simple, requiring standard molecular techniques, such as PCR, or adherence to step-by-step protocols. However, as an end-to-end protocol, it is intensive and ideally undertaken by experienced laboratory workers. The workflow needs to accommodate constraints on working hours and fit with other workloads to enable routine implementation. Realistically, the estimated 15-hour workflow for sample-to-sequence (for a batch of 24 samples) should be conducted over a 2–3 day period, but could be performed rapidly when required. Feedback from our training workshops indicated that hands-on training was valued and provided an appreciation of what it takes to produce quality sequence data. However, while useful as an introduction to MinION sequencing, workshops should be followed by extended one-on-one training and continued use to achieve full competency in performing the workflow.

### Challenges

Sub-optimal storage due to frequent power cuts and limited local storage facilities are a challenge in LMICs. Previously we have encountered difficulties in WGS of RABV samples archived in laboratories under variable conditions for up to a decade due to RNA damage ( Brunker *et al.*, 2015). We found that the amplicon-based approach ensured not only efficient viral read throughput on the MinION but also that samples varying in quality could be sequenced in the same way and with the best chance of success (a worst-case scenario approach). Generating longer amplicons for RABV sequencing ( Nadin-davis *et al.*, 2017) has proven difficult for samples typical of these conditions (K. Brunker, unpublished report) but may be an option for *in situ* sequencing when samples are fresh. We were unable to perform quantitative reverse-transcription PCR (qRT-PCR) on samples during field trips due to lack of equipment, but if available, this could inform the sequencing approach and aid as a diagnostic tool ( Marston *et al.*, 2019). In addition, sequencing can usefully validate diagnostic results, e.g. a cat sample in the Philippines was determined positive by dRIT but shown to be negative during sequencing preparation (K. Brunker, unpublished report). Notes from the dRIT on this sample indicated it had been difficult to interpret, therefore the sequence information provided vital data to confirm the sample had been misdiagnosed as a false positive.

More generally, logistical challenges impact on infectious disease research in LMICs, from sample collection difficulties to limited internet and reliable electricity supplies and sourcing of reagents and consumables. A recent study found only two thirds of hospitals providing surgical care in 21 LMICs have a continuous electricity source or an available generator ( Chawla *et al.*, 2018), problems that only worsen in more remote areas. To overcome these issues, we used a lab-in-a-suitcase approach, with portable equipment to enable quick setup in different locations using battery power if required. However, maintaining a cold chain was problematic. Access to generator backed -20°C (for reagents) and -80°C (RNA storage) freezers was limited, even in research facilities, and minor issues often taken for granted in UK labs (e.g. access to ice for benchwork with reagents) were common. ONT have recently developed a field version of some of their sequencing kits, containing lyophilised reagents to mitigate cold chain constraints. Lyophilised reagents could be an option for future work but are expensive and would require significant bulk purchasing to be affordable. Moreover, few local suppliers can provide sequencing-specific reagents and a considerable mark-up is added to already monopolised prices in research-rich countries like the UK/USA. Ordering directly from the company comes with its own difficulties, including expensive shipment costs, import taxes and airway bills for the delivery of reagents under cold-chain conditions. Delays at customs can compromise reagent viability and occurred frequently on our sequencing trips. To promote in-country scientists and genomic research, better solutions are needed for sustaining a reliable, affordable supply chain for reagents and consumables.

Bioinformatics and data management proved to be a bottleneck in our pipeline and are a primary target for optimisation. The lab-on-an-SSD method (ARTIC network) simplified the installation of bioinformatic software and available pipelines, but as this is a developing resource, we did encounter issues that stalled analysis. This was largely due to limited experience and lack of access to a dedicated bioinformatician to troubleshoot problems. In general there is a critical need for bioinformatics training in LMICs, which has been recognised as a priority area for development, e.g. the Pan African Bioinformatics Network for the Human Heredity and Health in Africa consortium and NIH-funded Application of Genomics and Modelling to the Control of Virus Pathogens (GeMVi). Future work will focus on support for bioinformatic pipeline development, including optimising barcoding assignment and consensus building thresholds for RABV samples. This may involve the integration of Read Assignment, Mapping, and Phylogenetic Analysis in Real Time ( RAMPART), a promising new tool to assess genome coverage and reference matching for each barcode concurrently as sequencing proceeds. We sequenced completely offline to avoid problems with internet connection (normally necessary to initialise MinION sequencing) and limit reliance on data transfer to servers for data storage. Sequence data was stored locally on either internal or external hard drives, with subsequent transfer to university servers in the UK at a later date. However, data transfer was problematic with the large number of files produced (in one instance corrupting a hard drive) and using one laptop for all sequencing and subsequent analysis was slow. Identifying local or cloud-based data solutions is advised to ensure data backup and take advantage of high-performance computing resources when available, but will come at additional cost.

RNA extraction from brain tissue was time-consuming and typically yields a low viral proportion after depletion of host genomic DNA ( Marston *et al.*, 2013). It also remains one of the most costly aspects of the workflow at ~£6 per sample. Goharriz *et al.* (2017) have shown that complete viral genome sequence can be obtained from Whatman Flinders Technology Associates (FTA) card samples without RNA extraction, which opens avenues for refining this part of the protocol and simplifying sample field collection and storage. Efforts to sequence from FTA card samples in Tanzania have shown promise, producing partial genome data (K. Brunker, unpublished report), and may be an option in future for field surveillance.

There is potential for cross-sample contamination, particularly when working with amplicon sequencing. This can occur both during library preparation and the bioinformatic stage of barcode demultiplexing following sequencing ( Xu *et al.*, 2018). As advised in Quick *et al.* (2016), we took precautions to minimise amplicon contamination in pre-PCR areas, reagents and equipment, such as physical separation between pre- and post-PCR areas, regular decontamination and aliquoting of reagents. We also processed and sequenced negative controls (in most runs) to assess the degree of contamination, finding a low level of amplicon contamination in most sequencing runs. However, the proportion of reads in the negative control relative to the total number of reads per run was low (median [range]=0.03 [0-1.5]%) for runs prepared outside of training workshops i.e. in normal working conditions. Contamination was most problematic in regions where products overamplified in certain samples, increasing the risk of cross-over to other samples. This could be mitigated by limiting the number of PCR cycles for high viral titre samples, if they can be assessed by qPCR prior to sequencing. Unsurprisingly, contamination was higher during training exercises (see underlying data; Brunker, 2019b) and any sequencing results from training should therefore be repeated for validation.

PCR bias may preferentially amplify specific haplotypes in a sample ( Grubaugh *et al.*, 2019). This has implications for the use of this type of data in intra-host diversity studies and our results indicate it can contribute to consensus sequence variation between platforms/approaches. Our comparisons between samples sequenced on different platforms (MinION vs Illumina) and with different methodology (PCR vs metagenomic) indicate that intra single nucleotide variation (iSNV) can change the nucleotides that hold the majority frequency in RABV consensus sequences. The extent to which this occurs may be influenced by the frequency of primer mismatches, depth of coverage and initial viral input ( Grubaugh *et al.*, 2019). Minor consensus variation has no impact on our objective of using phylogenetic-based methods to identify variants and spatio-temporal-genetic patterns. However, it does indicate that this sequencing approach will require some adjustment to accommodate in-depth analysis, such as inferring who-infected-whom and measuring intra-host diversity. Grubaught *et al.*’s experimental approach to “correct” such biases in amplicon-generated sequence data could be applied to limit false positive consensus assignments and accurately estimate within-host diversity in future.

The main limitation of an amplicon-based approach is the requirement for prior knowledge of the pathogen for targeted sequencing. This can be an issue if little is known about circulating RABV diversity in an area e.g. following an emergence event, an incursion of a new variant or where multiple divergent lineages co-circulate. We have found that primer design can accommodate a reasonable level of RABV diversity, but its application is still limited. Recent discoveries of novel lyssaviruses ( Shipley *et al.*, 2019), cross-species transmission events ( Kuzmin *et al.*, 2012; Mollentze *et al.*, 2014a) and the emergence of new variants ( Kotait *et al.*, 2019) indicate that when an amplicon-based approach is insufficient, a less targeted method would be useful to monitor threats as dog-mediated rabies is eliminated. Metagenomic sequencing is considered the gold standard for viral pathogen sequencing, offering an unbiased, untargeted approach but suffers from sensitivity issues when working with low viral titres. Success with RABV metagenomic sequencing varies according to sample quality and viral titre, with often <1% of reads mapping to a RABV reference sequence ( Brunker *et al.*, 2015). Therefore, a high sequencing throughput per sample is required. The MinION is not considered a high-throughput platform but recent and ongoing improvements to flowcell chemistry and data output from the MinION (10–20Gb in 48hours) have advanced the feasibility of conducting whole genome viral metagenomic sequencing directly from clinical samples, even with low viral titre samples ( Kafetzopoulou *et al.*, 2018).

### Opportunities

The value of genomic data cannot be understated and has yet to reach its full potential for RABV. While the emphasis of this paper is on the application of the workflow and not phylogenetic analysis, the genomic data has already provided new insights. For example, uncovering the presence of two RABV subclades in Kenya, including one that is closely related to the lineage found in Tanzania. The extent of cross-border transmission and the scale over which persisting RABV lineages circulate in East Africa will have important implications for national and regional control efforts. In Box 1, we suggest how genetic data can inform control measures at different stages in the pathway to elimination. In addition, during the application of our workflow in Tanzania we were able to show directly how genomic data enhanced an investigation of a rabies outbreak on the island of Pemba (K. Lushasi, Ifakara Health Institute, unpublished report). Pemba is a small island (988 km ^2^) off the coast of Tanzania. After four years of sustained vaccination campaigns, rabies appeared to have been eliminated from Pemba, with the last case detected mid-2014. However, after a lapse in vaccination efforts there was a rabies outbreak in 2016–2017. Samples collected in the early stages of the outbreak were exported to the UK as a priority for sequencing but still experienced a lag time of around three months. During our first field trip to validate in-country MinION sequencing in Tanzania (2017), we had the opportunity to sequence more samples from the outbreak. Eight samples were sequenced with a turnaround of two days, yielding whole genome consensus sequences that provided evidence of multiple incursions from mainland Tanzania (see Figure 7). This emphasises the importance of maintaining vaccination coverage and continued surveillance on Pemba to contain incursions and sustain freedom from rabies.

Box 1. **Surveillance insights from genomic data**

**Figure 8.**
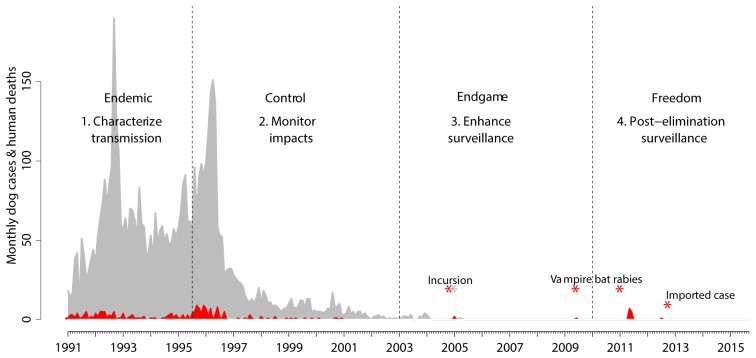
Progress towards elimination of dog rabies and role for genomic surveillance. Detected animal rabies cases are shown in grey and human rabies cases in red, based on surveillance data from Ecuador (from SIRVERA). Data were used to inform hypothetical scenarios relevant to rabies circulation in Latin America to illustrate examples of how genomic surveillance can add value. Stages of genomic surveillance corresponding to Box 1 are shown with examples of inferences from genomic surveillance indicated by asterisks.

Here we highlight the current and prospective ways in which genetic data can be used to answer questions at different stages along the complex pathway towards elimination of rabies ( Figure 8).

1. Characterize circulating variants and lineages:
  a. Identify host associations/biotypes to inform targeted vaccination, such as vampire bat versus dog rabies in Latin America ( Kotait *et al.,* 2019) or mongoose versus dog rabies in parts of Southern Africa ( Nel *et al.,* 2005), and the identification of domestic dogs as the reservoir host in a wildlife-rich ecosystem ( Lembo *et al.,* 2008)
  b. Assess lineage distributions in the context of political/administrative borders, such as provinces in the Philippines ( Tohma *et al.,* 2016), states in North Africa ( Talbi *et al.,* 2010) and East Africa ( Brunker *et al.,* 2015); and quantifying transboundary risks as shown by Trewby *et al.,* 2017.
  c. Determine the socio-ecological processes underlying local and long-distance transmission, for example the identification of long-distance human-mediated movement of RABV lineages ( Brunker *et al.,* 2015; Denduangboripant *et al.,* 2005; Trewby *et al.,* 2017) or associations with human density and landscape accessibility ( Dellicour *et al.,* 2017; Dellicour *et al.,* 2019).
2. Monitor progress of control:
  a. Report emergence and extinction of lineages as evidence of control failures or successes ( Dudas *et al.*, 2017)
  b. Integrate with data streams (e.g. case detection, bite patients, vaccination coverage) to resolve complexities in dynamics ( Bourhy *et al.*, 2016; Lembo *et al.*, 2008; Viana *et al.*, 2016)
  c. Identify epidemiologically connected areas that RABV lineages circulate between once control underway e.g. RABV spread associated with road networks, ( Brunker *et al.,* 2018; Talbi *et al.,* 2010), spread between peri-urban and rural areas ( Bourhy *et al.,* 2016).
3. Enhance surveillance during endgame:
  a. Differentiate undetected endemic transmission versus incursions ( Bourhy *et al.*, 2016; Faria *et al.*, 2017; Zinsstag *et al.*, 2017)
  b. Discriminate variants to verify freedom from dog rabies ( Velasco-Villa *et al.,* 2017) or identify emergence of new reservoirs, such as those seen in Latin America (primate/marmoset associated variants ( Kotait *et al.,* 2019), circulation in crab-eating foxes ( Carnieli *et al.,* 2009), terrestrial carnivores associations in Mexico ( Velasco-Villa *et al.*, 2005))
  c. Exclude possibility that cases are due to vaccine reversion, given vaccine quality concerns in some settings ( Suseno *et al.*, 2019)
4. Post-elimination surveillance:
  a. Identify the origin of imported cases (animal or human) or new outbreaks to direct control and prevention efforts and limit spread ( Mollentze *et al.*, 2013; Zhang *et al.*, 2019)

WGS using portable nanopore technology is a rapidly moving field, with protocols and pipeline developments evolving quickly. Although this comes with the caveat that protocols have to evolve to keep up with changes, these developments bring cost savings and improve accuracy. New refinements are expected to reduce costs further from a current estimate of ~£60 to £35 per sample, according to projections with flowcell re-use or the smaller low cost Flongle flowcell. Moreover, ongoing optimisation to reduce reagent use (K. Brunker, unpublished report) indicate that this can be reduced even further to the point where sample preparation/RNA extraction, rather than sequencing itself, become the most costly aspect of the protocol. Improvements to pore chemistry, kits and basecalling software have significantly improved the error rate, often considered the MInION’s Achilles heel. Single read accuracy has improved from 20% error rate of early phase 2014 releases to <5% error rate with R9.4.1 pore chemistry ( Plesivkova *et al.*, 2019). The latest version shows further improvements with 1D ^2^ sequencing technology and error correcting algorithms reaching ~99.3-99.9% consensus accuracy ( Wick *et al.,* 2019). A major benefit of nanopore sequencing is the ability to convert raw data to basecalled sequence data in real-time. Although we did not have a GPU powered laptop to take advantage of this function, it offers an even speedier resolution for time-critical sample analysis to inform decision-making. ONT’s MinIT, a portable data processing unit, offers similar functionality but with the flexibility to use with different laptops.

## Conclusions

Genomic research has the potential to leap-frog LMICs to the forefront of infectious diseases surveillance and control, and facing challenges head-on will hugely benefit the future of public and veterinary health. Most countries with endemic dog-mediated rabies currently have limited local sequencing capacity. These countries also suffer a heavy burden of other neglected endemic diseases, that could benefit from genomic research. The approach that we present is affordable and readily deployable to such settings with limited infrastructure, overcoming issues that typically constrain routine genomics surveillance, including cold chain, electricity and internet. Moreover, the approach is scalable and low-cost (~£60 per sample) for both sequencing large numbers of samples and for rapid turnaround to inform disease management. We predict further improvements to sequencing performance including cost-effectiveness, speed and accuracy, with further optimization underway and protocols available to researchers ( https://github.com/kirstyn/rabies_minion; Brunker, 2019d; Brunker, 2019e). Nonetheless, improvement to the supply and affordability of reagents for LMICs’ scientists is an area that needs addressing, as is the development of bioinformatics resources and translation of genomic research findings to digestible recommendations for policy-makers. Overall, this in-country application can support capacity building and the sustainability of routine genomic surveillance in LMICs, empowering local scientists to conduct cutting-edge research in their own backyard.

## Data availability

### Underlying data

Consensus sequences on Genbank, Accession numbers MN726802 - MN726882

Figshare: rabv_minionSeqConsensus.fasta. https://doi.org/10.6084/m9.figshare.10282859.v1 ( Brunker, 2019a)

Figshare: Table 1. Rabies virus samples sequenced on the MinION machine 2016–2019 in East Africa, Philippines and UK. https://doi.org/10.6084/m9.figshare.11111771.v1 ( Brunker, 2019b)

Figshare: Breakdown of costs to sequence rabies virus samples on MinION (spreadsheet). https://doi.org/10.6084/m9.figshare.11120405.v1 ( Brunker, 2019c)

### Extended data

Figshare: RabiesVirus_sampleToSequence_SOP. https://doi.org/10.6084/m9.figshare.10742090.v1 ( Brunker, 2019d)

Figshare: Rabies virus primer schemes. https://doi.org/10.6084/m9.figshare.11112134.v1 ( Brunker, 2019e)

Figshare: Extended Figure 6 phylogeny including tip labels: https://doi.org/10.6084/m9.figshare.12284006 ( Brunker, 2020a)

Figshare: Extended Figure 7 phylogeny including tip labels: https://doi.org/10.6084/m9.figshare.12284009 ( Brunker, 2020b)

Data are available under the terms of the Creative Commons Attribution 4.0 International license (CC-BY 4.0).

## Consent

Written consent to publish the images included in this paper was obtained from the individuals in these images.
