# Supplementary material for: Rapid in-country sequencing of whole virus genomes to inform rabies elimination programmes
Source: Wellcome Open Res. 2020 May 19;5:3. Originally published 2020 Jan 7. [Version 2] doi: 10.12688/wellcomeopenres.15518.2 (PMC7001756; doi:10.12688/wellcomeopenres.15518.2)
Supplement: Peer review history

# Peer review: Reviewer response for version 1

**DOI:** 10.21956/wellcomeopenres.16987.r37504

**Published:** 2020-02-10

**Authors:** Christine Carrington

**Affiliations:** 1 Department of Preclinical Sciences, Faculty of Medical Sciences, The University of the West Indies, St. Augustine, Trinidad and Tobago

Brunker *et al.* have presented a very detailed and thorough description of an end-to-end workflow for generation and bioinformatic analysis of rabies virus whole genome sequences using Nanopore technology and its validation in resource-limited settings. By including in-country training and involving local scientists in their validation they demonstrated the feasibility of the approach under realistic conditions and were able to identify typical challenges. The manuscript is very well written and the level of detail provided in describing the methods (e.g. the specifications for the computer used) is appreciated. The work presents a strong case for the use of in-country sequencing to inform rabies elimination programmes. I have only a few suggestions / comments:

1. Page 11 - The authors mention that workshop participants (presumably all 21) were asked to submit research proposals and commitment from their institutions to support MinION genomic work in order to obtain MinION starter kits. They then state that four MinION starter kits were provided to groups "to kickstart the application of their new training skills for genomic research". To provide readers with a better idea of the impact of the training, it would be helpful to know how many of the participants actually submitted proposals (with institutional commitment) as well as how many "groups" received kits. Also, is there information on whether any of the proposed projects have been initiated? 
2. Figure 5 - Although it is indicated in the table within Figure 5a and highlighted in the phylogeny in Figure 5b, I would suggest including the name of the Tanzanian whole genome sequence in the figure legend i.e. "...for a Tanzanian RABV whole genome sequence (tvla1_tz_Rab16031);"
3. Figure 6 - It would be useful to know the regions from which the Genbank sequences in the phylogeny originated (do they overlap with any of those sampled in the study?). I would also like to see a fully annotated tree (perhaps as extended data) that included bootstrap values, sequence IDs and the regions and years associated with all the sequences.
4. Figure 7 - As for Figure 6 a fully annotated tree (as extended data) would be a helpful addition.
5. Figure 8 - It is unclear whether the data in this figure is hypothetical or real data from a specific country? Please clarify in the Figure title or legend.

Is the rationale for developing the new method (or application) clearly explained?

Yes

Is the description of the method technically sound?

Yes

Are the conclusions about the method and its performance adequately supported by the findings presented in the article?

Yes

If any results are presented, are all the source data underlying the results available to ensure full reproducibility?

Yes

Are sufficient details provided to allow replication of the method development and its use by others?

Yes

Reviewer Expertise:

virology, RNA viruses, vector-borne viruses, virus evolution

I confirm that I have read this submission and believe that I have an appropriate level of expertise to confirm that it is of an acceptable scientific standard.

# Peer review: Reviewer response for version 1

**DOI:** 10.21956/wellcomeopenres.16987.r37507

**Published:** 2020-02-03

**Authors:** Herve Bourhy

**Affiliations:** 1 Unit Lyssavirus Epidemiology and Neuropathology, WHO Collaborating Centre for Reference and Research on Rabies, Institut Pasteur, Paris, France

Brunker *et al.* present an interesting paper describing an approach that can be used under minimal laboratory infrastructure and that could reduce the lag time from sample to sequence and epidemiological action in LMICs. They propose an in-country pipeline for rapid whole genome sequencing (WGS) of rabies viruses (RABV), one of the major neglected tropical disease causing a large public health and economic burden in Asia and Africa. The approach is based on the MinION sequencing technology, including a workflow from sample to sequence and the associated bioinformatics pipeline.

 The paper is well written and documented. The authors provided a lot of information and incorporate procedures for downstream analysis, interpretation, and management of genomic data in a surveillance context. The feasibility of this methods was tested in different locations in Kenya, Tanzania and the Philippines using the concept of a lab-in-a-suitcase platform:

 To this aim, samples from Kenya (n = 10), Tanzania (n = 24) and the Philippines (n = 52) were sequenced using three distinct technologies and 92 of them were sequenced in LMICs using the MinION sequencing technology. The protocol included RNA extraction and PCR amplification of 400bp amplicons across the entire genome (~11923bp), with an overlap of 50bp. Therefore, at least 30 different amplicons were needed to cover the whole RABV genome. The costs per sequenced sample were estimated for the MinION-based multiplex PCR approach and compared favorably with the previously used Illumina-based metagenomic approach. Therefore, the authors claimed that they demonstrate the feasibility of real-time sequencing of RABV to rapidly inform policy decisions and disease management. I would easily agree that the first objective is reached and I really consider that as a great achievement which deserves publication. But this does not seem to be fully the case for the second one (inform policy decision and disease management). Most of the limitations of the techniques are presented in the discussion. However, some of the limitations would require more discussion and others are simply not addressed at all although some of these dramatically weaken the ability of this approach to accurately inform epidemiological surveillance and complex epidemiological questions.

 There are many studies published by other authors showing that samples collected in a defined geographic area reflecting an endemic situation and during a limited period of time (a few months) differ only by a very low number of SNPs. Any errors concerning the distribution of these SNPs along the genomes and between the samples will have a dramatic effect on the phylogenetic tree reconstruction and the derived inferences.

 As mentioned by the authors, the MinION is so far not considered a high-throughput platform. One of the major risks is the potential for cross-sample contamination, particularly when working with amplicon sequencing. This was noticed by the authors “A low level of cross-contamination was noted in all sequencing runs (a few hundred reads), often caused by amplicons with particularly high levels of amplification”. This can occur both during library preparation and the bioinformatic stage of barcode demultiplexing following sequencing as written by the authors but the major risk lies in the preparation of the samples and their amplification. The authors stressed the fact that they took precautions to minimise amplicon contamination in pre-PCR areas, reagents and equipment. However, all clinical biology laboratories even in the developed countries have shifted to single tube amplifications to avoid these contaminations. It appears unrealistic to think that such contaminations will not happen in a significant percentage when performed in field conditions when many samples will be received and processed every day and at least 30 different amplicons generated for each of them. This will unfortunately happen independently of all authors’ efforts to train local people that are documented in this manuscript.  These cross contaminations and mislabelling of tubes will have a strong effect on the final whole genome sequences generated and consequently on some of the conclusions drawn from their phylogenetic analysis.

 I fully agree with the fact that improvements to pore chemistry, kits and basecalling software have significantly improved the error rate of the MInION. However, despite these improvements every user will agree that when used in the field condition the consensus accuracy reach only 98-99% which seems to be quite low for many applications.

 The authors are fair writing “Our comparisons between samples sequenced on different platforms (MinION vs Illumina) and with different methodology (PCR vs metagenomic) indicate that intra single nucleotide variation (iSNV) can change the nucleotides that hold the majority frequency in RABV consensus sequences. The extent to which this occurs may be influenced by the frequency of primer mismatches, depth of coverage and initial viral input”. This is further exemplified in Figure 4 where locations of single nucleotide polymorphisms between Illumina and MinION consensus sequences are shown (at least two on this example). However, I disagree with the rather superficial conclusion saying “Minor consensus variation has no impact on our objective of using phylogenetic-based methods to identify variants and spatio-temporal-genetic patterns”. This has to be demonstrated and is probably not the case for many of the applications of genomic data on surveillance. I would bet that the longer internal branches of the maximum likelihood tree linking the rabies virus genomes from Kenya and Tanzania to those collected from Genbank sequences illustrate these points. Further the authors should comment on the impact of the sites masked by ambiguity code N (coverage was <20X) and their final proportion on the full genome sequences.

 Two broad applications of the presented protocol are claimed by the authors such as characterization of circulating RABV diversity, and rapid in situ genomic surveillance of new cases, particularly during the endgame and I will agree that the error rate of the MinION will generally not interfere too much with such applications. However later in the discussion they presented many more applications of genetic data to inform surveillance (see Box 1, page 15: Current and prospective ways in which genetic data can be used to answer questions at different stages along the complex pathway towards elimination of rabies). Among the four main applications listed by the authors and their secondary objectives (10 are cited), at least 6 of them (listed below) will not tolerate cross contamination, ambiguities in the sequencing, and sequencing errors even in a very low percentage and therefore renders the proposed pipeline inapplicable for such studies.

- Identify host associations to inform targeted vaccination.
- Assess lineage distribution in the context of political/administrative borders, quantifying transboundary risks.
- Determine the socio-ecological processes underlying local and long-distance transmission.
- Resolve complexities in dynamics.
- Identify epidemiologically connected areas that RABV lineages circulate between once control underway.
- Discriminate variants to verify freedom from dog rabies or identify emergence of new reservoirs.

This should be made very clear in the discussion even if we may all share the author’s hope that further development in the technology will in the next years minimize the risk of sequencing errors.

 In conclusion, the MinION based pipeline proposed is interesting to enhance surveillance during the endgame and the post-elimination phases, stages that are not reached so far by many LIMCs, but is not appropriate for addressing complex epidemiological questions related to RABV dynamics and drivers of persistence and diffusion in an endemic or epidemic context.

Is the rationale for developing the new method (or application) clearly explained?

Yes

Is the description of the method technically sound?

Yes

Are the conclusions about the method and its performance adequately supported by the findings presented in the article?

Partly

If any results are presented, are all the source data underlying the results available to ensure full reproducibility?

Yes

Are sufficient details provided to allow replication of the method development and its use by others?

Yes

Reviewer Expertise:

NA

I confirm that I have read this submission and believe that I have an appropriate level of expertise to confirm that it is of an acceptable scientific standard.

# Peer review: Reviewer response for version 1

**DOI:** 10.21956/wellcomeopenres.16987.r37505

**Published:** 2020-01-27

**Authors:** Claude Sabeta

**Affiliations:** 1 OIE Rabies Reference Laboratory, Agricultural Research Council-Onderstepoort Veterinary Institute, Onderstepoort, South Africa

The authors Brunker *et al.* have described and advocate a rapid in-country sequencing of whole virus genomes to inform rabies elimination programmes using Tanzania, Kenya and the Philippines as sites of testing of this technology. Whereas genomic surveillance is not being used in Africa and Asia and as we know this is due to limited infrastructure, costs, supply chains and other logistical challenges. This may change as the global scientific community has adopted the zero human deaths by 30. This may result is a shift in funding for this elimination programme as regional blocks may support this programme to be in line with global goals for the elimination of rabies. With this surveillance approach, regional members will be able to differentiate rabies variants from other wildlife, distinguish ongoing transmissions from incursions as well as identifying sources of incursions. The authors described sequencing and bioinformatics pipelines and validation process. Protocols for sample-to-sequence and a general purpose for analysis, interpretation and management of rabies virus genome data are available making the approach described here appealing to regional laboratories and members states. The concept of a portable, low-cost sequencer (lab-in-a suitcase is interesting). Overall, the manuscript is well written and I have very few issues that require clarification:

1. Para 4 - please explain what is meant by disease can circulate for extended periods at low levels in the context of rabies.
2. In many of these countries, there are rabies virus repositories that date to many years back. What would the authors such laboratories in terms of sequencing to inform rabies elimination programmes?
3. The samples were collected by field officers, in actual fact a varied number, Kenya=10, Tanzania=24 and the Philippines=52. Do these numbers in any way indicate how good the different countries are in collecting and submitting samples? If so, how can this be improved so that whole genome sequencing of viruses truly reflects the rabies situation in the countries?
4. On page 5, the authors indicated that samples were processed in settings with varying infrastructure and resources - samples were confirmed using different methods from RIDT, to dRIT and the FAT. Can the authors shed more light on the antibody preparation used in the dRIT and also the FAT in the Philippines? What was the correlation between the results obtained by these laboratories and the sequencing?
5. In the description of the Trizol method on page 5 - 50-100g should read 50-100mg.
6. Page 7 - please indicate the number of archived and tested samples to give the reader a sense of the sample numbers.
7. On page 7, the authors indicated that extracted RNA was reverse transcribed first with the Protoscript II kit and later Lunascript RT Supermix - was the rationale only to use reduced volumes or they were other considerations?
8. I think the technology described in this manuscript is useful for the Asia and Africa, particularly as the global community move towards rabies elimination human mediated dog rabies. Some of the outputs have been taken up by Kenya, Tanzania and the Philippines. How do the authors intend to disseminate such important technologies (particularly on minimising cross-contamination in sequencing runs, preparation of sequencing libraries and bioinformatics training) for this purpose to other regions of the two continents (if any)?
9. On page 10 - one salivary gland sample (amend).
10. Page 11 - Variations in quality of brain tissues was observed and generally influenced sample preparation times - what would the authors advise on sample collection in general to overcome this problem?
11. How do the authors plan to overcome the bottlenecks experienced with bioinformatics and data management?
12. The authors indicated that prior knowledge about circulating RABV diversity in an area is important - and we know many countries on the African and Asian continents do not have this knowledge. How do the authors hope that member states on these continents can circumvent this issue?

Is the rationale for developing the new method (or application) clearly explained?

Yes

Is the description of the method technically sound?

Yes

Are the conclusions about the method and its performance adequately supported by the findings presented in the article?

Yes

If any results are presented, are all the source data underlying the results available to ensure full reproducibility?

Yes

Are sufficient details provided to allow replication of the method development and its use by others?

Yes

Reviewer Expertise:

Epidemiology, virus evolution, virus diagnostics, method development and validation.

I confirm that I have read this submission and believe that I have an appropriate level of expertise to confirm that it is of an acceptable scientific standard.
